# Front‐line treatment patterns in multiple myeloma: An analysis of U.S.‐based electronic health records from 2011 to 2019

**DOI:** 10.1002/cam4.4137

**Published:** 2021-08-16

**Authors:** Shaji Kumar, Mellissa Williamson, Uzor Ogbu, Andy Surinach, Stella Arndorfer, Wan‐Jen Hong

**Affiliations:** ^1^ Division of Hematology Mayo Clinic Rochester MN USA; ^2^ Genentech, Inc. South San Francisco CA USA; ^3^ Genesis Research Hoboken NJ USA

**Keywords:** multiple myeloma, prescribing trends, treatment outcome

## Abstract

Multiple myeloma (MM) treatment options have evolved rapidly, but how new agents are incorporated into treatment decisions in current practice is not well understood. This study examined prescribing trends of physicians treating newly diagnosed MM and treatment outcomes in the United States. Electronic health record data from 6271 adult patients diagnosed with MM and receiving initial treatment between 1 January 2011 and 31 January 2020 were derived from the Flatiron Health electronic‐health record de‐identified database. The number/types of agents included in therapy regimens, time to next treatment (TTNT), and overall survival (OS) were assessed. Subgroups were analyzed by the International Staging System (ISS) disease stage at diagnosis, stem cell transplant eligibility and timing, and practice type. Exploratory prognostic models evaluated the association between baseline covariates and time‐to‐event outcomes. The proportion of patients receiving triplet therapies increased from 2011 (36%) to 2019 (72%) as those receiving initial monotherapy or doublet therapy decreased. Overall, the most prevalent triplet regimen consisted of an immunomodulatory drug (IMiD), a proteasome inhibitor, and a steroid. From 2017 to 2019, median TTNT from front‐line to second‐line was longer in patients with ISS stage I versus stages II/III, and in those receiving IMiD‐containing doublet or triplet therapies versus other combinations. Overall median OS was 56 months and increased from 2011 to 2014, after which median OS was not yet reached. Age, ISS stage, and high‐risk status were prognostic for both OS and TTNT, while sex, practice type, and ECOG status were prognostic for OS only.

## Graphical abstract

This manuscript is a retrospective study based on US health records representative of the standard real world approach to treatment of myeloma. The study identifies the gradual shift in the treatment approaches to newly diagnosed myeloma, with increasing use of triplet regimens, and the improved survival outcomes with the current approaches.

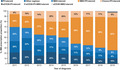

## 1 INTRODUCTION

Treatment options for multiple myeloma (MM) have evolved significantly over the past 5–10 years, with several effective new agents introduced and many more under investigation. For front‐line therapy, the availability of the novel drug class for MM, CD38‐targeting antibody (daratumumab), was approved for newly diagnosed MM patients in the United States (US) in 2018 and 2019 for patients who are ineligible and eligible for transplant, respectively. Survival rates have increased by 50% from 2004 to 2017, especially among the elderly.^1^ Moreover, patients who receive a transplant have longer median overall survival (OS) of 6.1 years versus patients who do not receive a transplant (median OS 4.0 years)^2^; however, despite these advances, MM remains incurable, with a 5‐year OS rate yet to reach 55%^3^ (77% stage I, 53% stage II, 19% stage III)^4^ and unmet needs persist in high‐risk, elderly, and minority patients.^5^

The chronic, progressive nature of MM often leads to multiple relapses, and several regimens are given throughout the patient's treatment course.^6^, ^7^ Currently available therapies may prevent or delay life‐altering complications when patients are responsive to them; however, toxicities and development of resistance limit their continued use.^8^, ^9^ Time spent on front‐line therapy, time to progression, and time to next treatment (TTNT) are associated with the depth of response to initial treatment, with regimens given for subsequent relapses associated with greater toxicity and likelihood of discontinuation.^7^ The National Comprehensive Cancer Network (NCCN) guidelines (v4.2020) recommend considering triplet regimens in patients who can tolerate them. However, these newer regimens have the potential to increase treatment‐associated toxicities, an important factor in determining which patients may receive them.^9^

The breadth and timing of treatment patterns for MM have been examined periodically; however, there is a need to understand the most current real‐world treatment practices and outcomes in this rapidly evolving environment.^7^, ^10^, ^11^, ^12^, ^13^ For example, the real‐world use of front‐line combination therapy with proteasome inhibitors (PI), immunomodulatory drugs (IMiD), and anti‐CD38‐targeting agents remains unknown.^9^

We report trends in front‐line treatment, TTNT, and OS for newly diagnosed patients with MM in the United States from 2011 to 2019 overall and by stage at diagnosis, and stem cell transplant (SCT) eligibility.

## 2 METHODS

### 2.1 Study population

**FIGURE 1:**
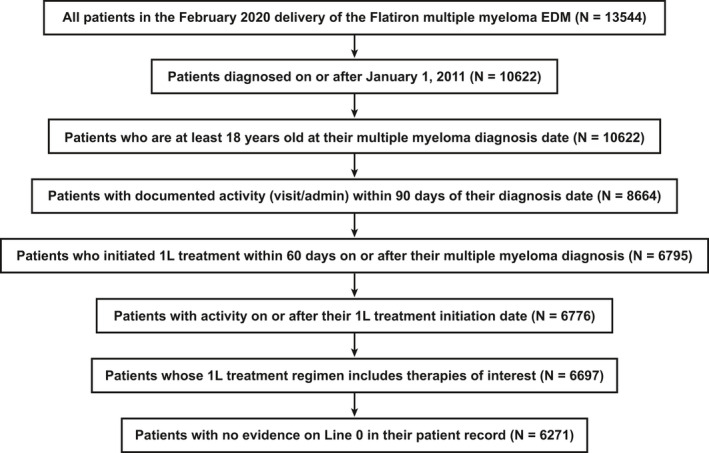
Patients with MM assessed and included in the analysis. 1L, front‐line; EDM, enhanced data mart; MM, multiple myeloma

This retrospective cohort study included patients ≥18 years old in the Flatiron Health Network who were diagnosed with MM (ICD‐9, 203.0x or ICD‐10, C90.xx) on or after 1 January 2011, and received front‐line treatment on or before 31 January 2020 and within 60 days of diagnosis (Figure 1). We report results through 31 December 2019 given that only 1 month of data is reported for 2020. The Flatiron Health database is a nationally representative, retrospective, longitudinal database derived from electronic health records (EHR) from >280 cancer clinics (approximately 800 sites of care), representing >2.4 million patients with active cancer in the United States. The Flatiron Health EHR‐derived database includes structured and unstructured patient‐level data that are processed through technology‐enabled abstraction (including physician notes, radiology/pathology/biomarker reports, and discharge summaries) and refreshed monthly. Data obtained from Flatiron Health were de‐identified, and provisions were in place to prevent re‐identification to protect patient confidentiality.

Notably, most patients in the database originate from community oncology settings; however, relative community/academic proportions may vary depending on the study cohort. All the academic cancer centers currently included in the Flatiron Health database are NCI‐designated cancer centers. These cancer centers are part of larger, multi‐specialty academic medical centers, which include both inpatient and outpatient facilities that are affiliated with a medical school and teaching hospital(s). Academic‐affiliated clinics, even those based in the community, are still categorized as “academic.”

Patients visited the clinic at least twice for their established MM diagnosis and began a recognized treatment regimen within 60 days of diagnosis. We excluded patients with unknown or incomplete treatment history, including those who started treatment before entering the Flatiron Health Network. Patients enrolled in clinical trials of investigational treatments were ineligible for inclusion in this analysis. All newly diagnosed patients who received an MM treatment recommended in NCCN guidelines were included in this analysis.

### 2.2 Variables and outcome measures

Treatment regimens were characterized in two ways: (1) according to the classes of drugs included in the regimen, including CD38‐targeting antibody, IMiDs, PIs, chemotherapy, histone deacetylase inhibitor, corticosteroids, other, and (2) by the number of agents in each regimen. Drugs and their associated classes are as listed in the NCCN Guidelines 2011 to 2019 for primary treatment of MM and are provided in Table S1. Each regimen was categorized as monotherapy (1 agent), doublet (2 agents), triplet (3 agents), or quadruplet (≥4 agents). The majority of these agents are administered in combination with corticosteroids, with corticosteroids counted as an agent in the regimen.

We investigated trends in front‐line prescribing by the drug classes included in each regimen and the number of agents in the regimen in the overall cohort of patients with newly diagnosed MM and among three subgroups of patients. The first subgroup consisted of patients with records that contained staging information at diagnosis. *MM stage at diagnosis* was based on a combination of “coded staging” contained in the Flatiron Health Database and “derived staging” using the most recent albumin and β_2_‐microglobulin test results within 60 days before or after the diagnosis date and before the start of treatment. If a discrepancy arose between the coded and derived staging methods, derived staging was used. Derived staging was based on the International Staging System (ISS; Table S2).^14^ In the second subgroup, *transplant*‐*ineligible patients* were defined as those aged ≥70 years at the time of MM diagnosis. In clinical practice, transplant eligibility is determined by several factors, including age, comorbidities, and other clinical considerations. However, this level of data was not available for this analysis; therefore, age was used as a proxy for transplant eligibility. Age at diagnosis was determined using the month and year of diagnosis, and the birth date was set to the 15th day of the month noted in the record. The third subgroup of *early transplant recipients* was defined as patients with a documented SCT within 12 months of the commencement of front‐line therapy, measured from the start date of front‐line treatment to the transplant date as noted in the medical record.

TTNT, described in months, was defined as the time from the start of front‐line therapy to the start of second‐line therapy. Patients with no recorded second‐line therapy were censored at death or last recorded activity in the medical record. Similarly, OS was defined as months between the start of front‐line therapy and death date or censor date.^15^ TTNT for front‐line treatment and OS were assessed in the overall population and by ISS disease stage at diagnosis and receipt of SCT. Exploratory prognostic models for TTNT and OS were developed to understand associations between baseline covariates and time‐to‐event outcomes within treatment groups.

### 2.3 Analysis

Descriptive analyses of prescribing trends based on the drug classes included in the regimen prescribed to each patient, and the number of drugs contained in each regimen, including steroids, were conducted.

A Kaplan–Meier analysis was used to determine the median TTNT and median OS, with 95% Brookmeyer–Crowley confidence intervals (CIs) constructed using the log of survival time. Prognostic models for TTNT and OS included the following baseline covariates: Eastern Cooperative Oncology Group (ECOG) performance status, age at diagnosis, high‐risk cytogenetics (including del(17/17p), t(4;14), t(14;16), t(14;20), and gain(1q)),^16^ ISS disease stage, sex, region, and practice type. Cox proportional hazards models were used to describe the association between baseline covariates and time‐to‐event outcomes. Hazard ratios and their corresponding 95% CI were reported for each covariate.

Statistical analyses were performed using R v4.0.0 (2020‐06‐30).

## 3 RESULTS

### 3.1 Patient demographics and characteristics

The Flatiron Health EHR‐derived database consisted of 13,544 patients with MM from which we identified 6271 front‐line‐treated patients who met the inclusion criteria (Figure 1). Patients were predominantly white (62%), with just over half of the population aged ≥70 years, which remained the same over the time period 2011 to 2019 (Table 1). The cohort contained slightly more men (55%) than women. Almost 60% of all patients were from the South (38%) and the Northeast (19%) regions and almost 70% were diagnosed between 2015 and 2019. Approximately 50% of patients had missing ECOG performance status, with the majority (>50%) with non‐missing values of 0 or 1. Disease stage at diagnosis was evenly spread across stages I to III, with stage I showing a steady increase over the time period 2011 to 2019; however, disease stage information was only available for 59% of patients in the cohort. Approximately 92% of patients received a cytogenetic test (including fluorescence in situ hybridization [FISH] and karyotyping) at any time during the study period, including del 17/17p, t(4;14), t(14;16), t(14;20) and chromosome 1 abnormality; testing frequency increased over time from 82% in 2011 to 93% in 2019. Approximately 30% of patients were classified as high‐risk, which increased during the study period from 15% in 2011 to 37% in 2019, mostly driven by chromosome 1 abnormality (~20%). The distribution of patients by practice type was 10% academic and 90% community; of the >20% of patients who received a transplant within 12 months of treatment, a higher proportion was seen in academic centers (17%) compared with transplant‐ineligible patients (7%). From 2011 to 2018, approximately 25%–28% of patients received SCT annually.

**TABLE 1:** Demographics and characteristics of the analysis cohort.

Characteristic, *n* (%)	Patients (*N* = 6271)
Age at diagnosis	
Age <70 years	3103 (49)
Age ≥70 years	3168 (51)
Sex	
Male	3431 (55)
Female	2840 (45)
Practice type where care received	
Community	5619 (90)
Academic	652 (10)
Year of diagnosis	
2011	301 (4.8)
2012	464 (7.4)
2013	576 (9.2)
2014	641 (10)
2015	745 (12)
2016	907 (14)
2017	831 (13)
2018	917 (15)
2019	852 (14)
ISS stage at diagnosis	
I	1249 (20)
II	1172 (19)
III	1293 (21)
Unknown/not documented	2557 (41)
ECOG performance status at diagnosis	
0	1145 (18)
1	1250 (20)
2+	675 (11)
Unknown/not documented	3201 (51)
Stem cell transplant, yes	1563 (25)
High‐risk^a^ cytogenetics, yes	1840 (29)
Deceased	2163 (34)

Abbreviations: ECOG, Eastern Cooperative Oncology Group; ISS, International Staging System.

^a^ Based on cytogenetic abnormality: del(17/17p), t(4;14), t(14;16), t(14;20), gain(1q) [chromosome 1 abnormalities].

### 3.2 Prescribing trends for front‐line MM treatment

In 2011, front‐line MM treatment regimens (*n* = 6271) were most often doublets (48%) or triplets (36%); however, by 2019, triplets accounted for the majority of front‐line regimens (72%) (Table 2), with a >60% reduction in the use of doublets. Monotherapy use has also substantially decreased over the time period 2011 to 2019 (~75% reduction), while quadruplets remained stable until 2017 after which a steady increase is shown.

**TABLE 2:** Front‐line MM prescribing trends by treatment regimen, 2011 to 2019.

	Front‐line MM prescriptions, *n* (%)								
	2011 (*n* = 301)	2012 (*n* = 464)	2013 (*n* = 576)	2014 (*n* = 641)	2015 (*n* = 745)	2016 (*n* = 907)	2017 (*n* = 831)	2018 (*n* = 917)	2019 (*n* = 852)
Monotherapy	40 (13)	70 (15)	59 (10)	65 (10)	51 (6.8)	50 (5.5)	30 (3.6)	52 (5.7)	29 (3.4)
Doublet	144 (48)	233 (50)	232 (40)	258 (40)	300 (40)	271 (30)	234 (28)	190 (21)	160 (19)
Triplet	108 (36)	159 (34)	270 (47)	303 (47)	376 (50)	562 (62)	543 (65)	642 (70)	617 (72)
Quadruplet	8 (2.7)	1 (0.2)	15 (2.6)	15 (2.3)	17 (2.3)	23 (2.5)	22 (2.6)	30 (3.3)	43 (5.0)

Abbreviations: MM, multiple myeloma.

**FIGURE 2:**
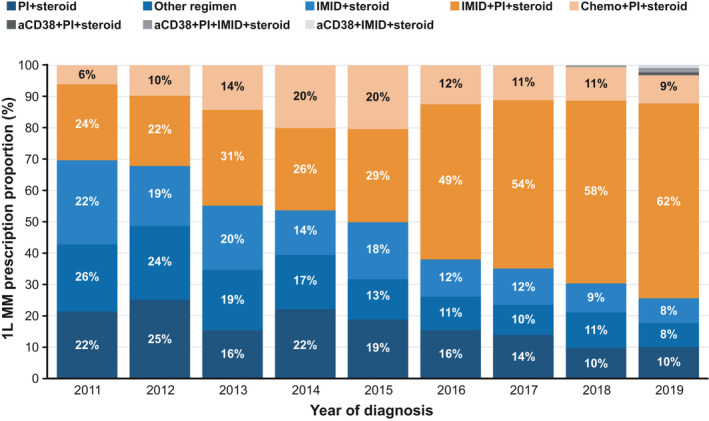
Prescribing trends for the most prevalent treatment regimens per year. The 1L MM prescription proportion for the combined aCD38 groups (aCD38 + PI + steroid, aCD38 + PI + IMID + steroid and aCD38 + IMID + steroid) were 0.1% for diagnosis in 2016, 0.1% for diagnosis in 2017, 0.4% for diagnosis in 2018 and 3% for diagnosis in 2019. 1L, front‐line; chemo, chemotherapy; IMiD, immunomodulatory drug; MM, multiple myeloma; PI, proteasome inhibitor

**FIGURE 3:**
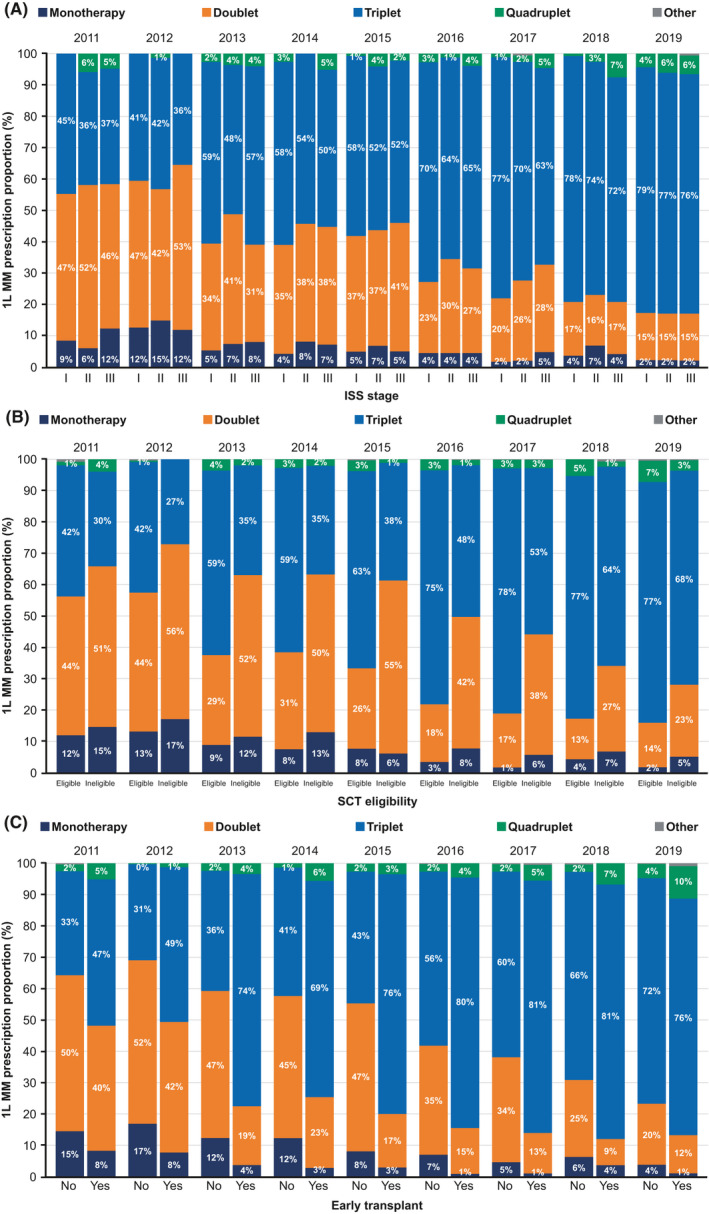
Treatment regimen prescribing trends by stage or SCT subgroup, 2011–2019. Observed proportion of prescriptions for monotherapy, doublet, triplet, or quadruplet 1L therapy by (A) disease stage at diagnosis, (B) SCT eligibility at diagnosis, and (C) receipt of SCT therapy within 12 months of diagnosis. Early transplant recipients were defined as patients with a documented SCT within 12 months of the commencement of 1L therapy, measured from the start date of 1L treatment to the transplant date as noted in the medical record. Percentages for “other” treatment are all <1. 1L, front‐line; ISS, International Staging System; MM, multiple myeloma; SCT, stem cell transplant

Overall, the most commonly used front‐line MM treatment regimen was IMiD + PI + steroid, which increased by over 2.5‐fold from 2011 to 2019, accounting for over 60% of all front‐line treatment prescriptions in 2019. Over this time period, the use of doublets decreased from 48% in 2011 to 19% in 2019 (Figure 2). The increase in triplet use among stage I patients, (less than 2‐fold) was not as steep compared to stages II and III which saw an increase of over 2‐fold from 2011 to 2019 with stage II patients having an almost 3‐fold increase in IMiD + PI + steroid triplet regimen (Figure 3A).

We also examined trends in treatment regimens based on patient transplant eligibility (*n* = 2743) and a history of early SCT (*n* = 1563). Overall, both transplant‐eligible patients and patients who received an SCT within 12 months of initiation of front‐line therapy (early transplant recipients) received triplet therapy more often than those who were ineligible for SCT (*n* = 3528) or who did not receive an early SCT (*n* = 1180) (Figure 3B,C). Between 2011 and 2019, transplant‐eligible patients received triplet therapy more than doublet therapy as front‐line treatment, whereas in transplant‐ineligible patients, a larger proportion of patients received doublet therapy from 2011 to 2015 after which a steady increase in triplet therapy was shown up to 2019.

### 3.3 Time to next treatment

**FIGURE 4:**
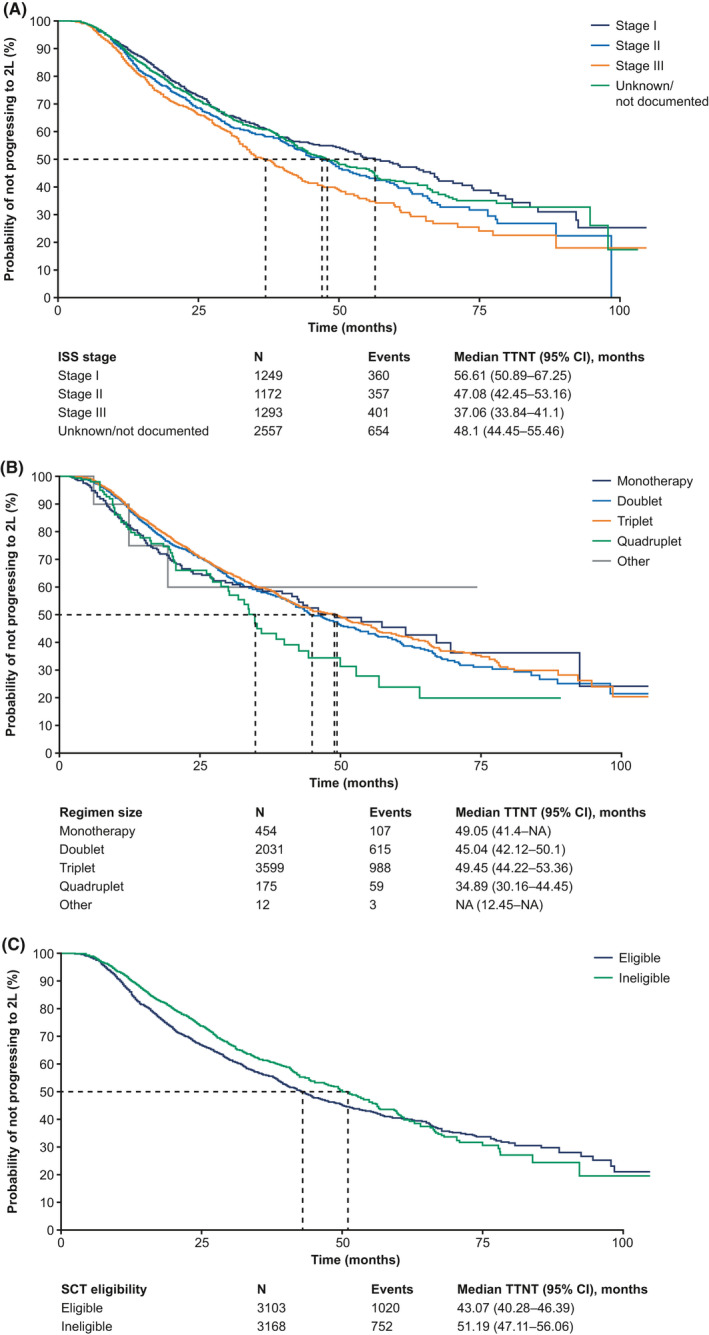
TTNT among patients with newly diagnosed MM by (A) disease stage at diagnosis, (B) regimen size, and (C) SCT status. Dashed line indicates the median TTNT. 2L, second‐line; ISS, International Staging System; MM, multiple myeloma; SCT, stem cell transplant; TTNT, time to next treatment

As of 31 December 2019, 1771 patients (28%) in the cohort had initiated second‐line treatment. The median TTNT (from front‐line to second‐line) was 46.7 months (95% CI, 44.0–50.1 months) during the study period. Median TTNT by disease stage was 56.6, 47.1, and 37.1 months for patients diagnosed with stage I, II, and III disease, respectively (Figure 4). Patients initiating quadruplet therapy regimens had the shortest median TTNT at 35 months compared with monotherapy (49 months), doublet therapies (45 months), and triplet therapies (49 months). Higher percentages of patients receiving quadruplet therapies were diagnosed at stage III disease and had high‐risk status compared to patients receiving monotherapy, doublets, and triplets. Patients receiving IMiD + steroid and IMiD + PI + steroid had the longest median TTNT at ~55 months followed by Chemo + PI + steroid and Other Regimen at ~40 months. Compared with the overall cohort, patients who received SCT had a median TTNT that was approximately 60 months, whereas among patients who did not receive SCT the median TTNT was 44 months (Table S3).

We conducted exploratory prognostic models to identify associations between baseline factors (including age at diagnosis, ECOG performance status, high‐risk status, ISS disease stage, sex, and practice type) and TTNT within treatment groups (Table 3). Overall, three variables were associated with a shorter TTNT (including ISS disease stages II and III and high‐risk status). A higher number of patients aged ≥70 years died after receiving front‐line therapy (*n* = 1019; 32%) compared to patients <70 years old (*n* = 401; 13%). Because patients with no recorded second‐line therapy were censored at death^15^ and more patients <70 years old were alive to receive second‐line treatment, age was not feasible to be considered as a candidate variable for the prognostic model for TTNT. ISS disease stages II and III and high‐risk status were significantly prognostic for shorter TTNT in early transplant patients. Moreover, female gender in early transplant recipients was prognostic for longer TTNT. In the transplant‐ineligible cohort, only high‐risk status was a significant prognostic factor for shorter TTNT.

**TABLE 3:** Overall prognostic model for TTNT.

Characteristic	HR	95% CI	*p* value
Age at diagnosis			
Age <70 years	—	—	
Age ≥70 years	1.13	1.01, 1.26	0.029
Received SCT	1.11	0.99, 1.24	0.078
Practice type			
Community	—	—	
Academic	0.92	0.62, 1.36	0.7
ECOG			
0	—	—	
1	1.03	0.88, 1.21	0.7
2+	0.96	0.86, 1.17	0.7
Unknown/not documented	0.97	0.85, 1.11	0.7
ISS stage			
Stage I	—	—	
Stage II	1.18	1.02, 1.37	0.027
Stage III	1.41	1.22, 1.63	<0.001
Unknown/not documented	1.12	0.98, 1.28	0.092
High‐risk status			
No	—	—	
Yes	1.45	1.31, 1.61	<0.001
Year of diagnosis			
2011–2015	—	—	
2016–2019	0.94	0.85, 1.05	0.3

Abbreviations: CI, confidence interval; ECOG, Eastern Cooperative Oncology Group; HR, hazard ratio; ISS, International Staging System; SCT, stem cell transplant; TTNT, time to next treatment.

### 3.4 Overall survival

**FIGURE 5:**
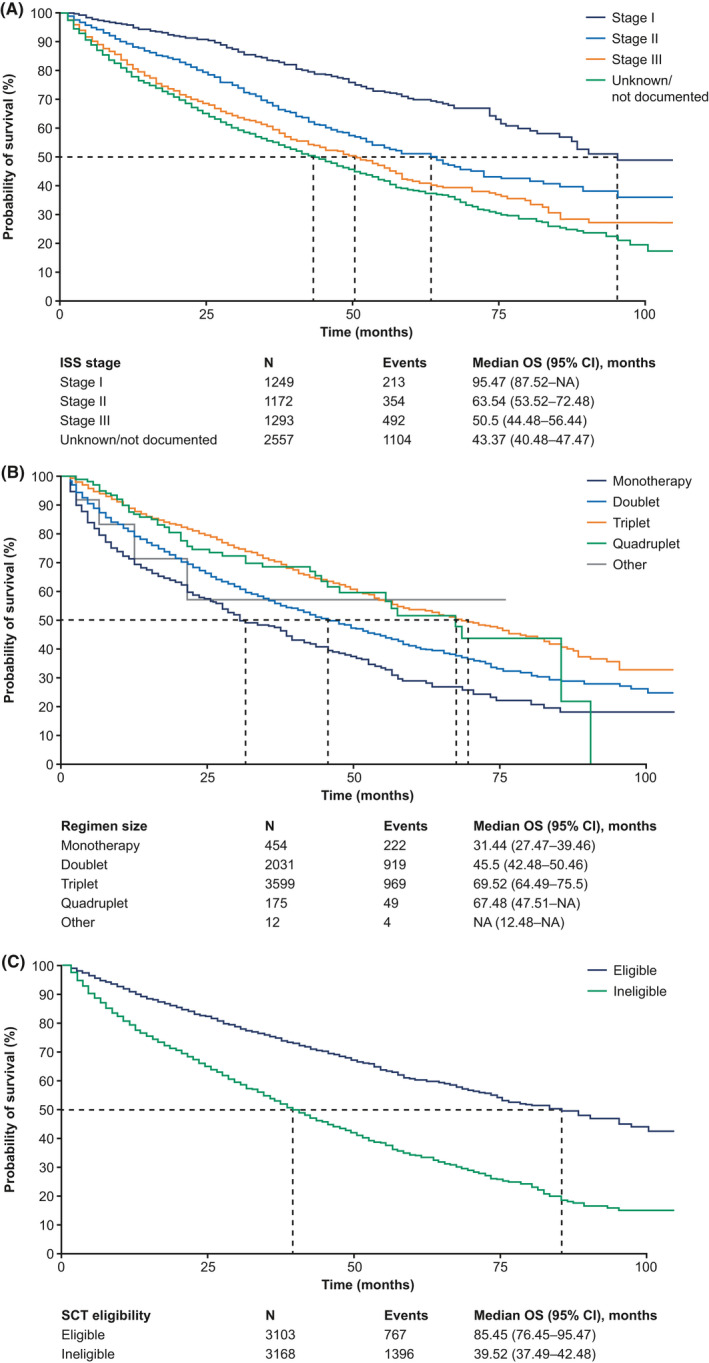
OS among patients with newly diagnosed MM by (A) disease stage at diagnosis, (B), regimen size, and (C) SCT eligibility. Dashed line indicates the median OS. ISS, International Staging System; MM, multiple myeloma; OS, overall survival; SCT, stem cell transplant

In this MM patient cohort, 2163 (34%) died or were censored as of 31 January 2020. The median OS was 56 months (95% CI, 54–58 months), with median OS decreasing with higher ISS stage (95 months, 64 months, 51 months for stages I, II, III, respectively) (Figure 5). Patients receiving triplet regimens had the longest median OS at 70 months, with patients receiving IMiD + PI + steroid having a median OS of 71 months, followed by those receiving quadruplet regimens at 67 months and those receiving doublets at 46 months. These broader trends are similarly seen for transplant‐ineligible patients, while over 80% of early transplant patients were still alive as of 31 January 2020. From 2011 to 2014, there was a 10‐month increase in median OS reaching 64 months in 2014, after which median OS was not reached in subsequent years up to 2019. A larger proportion of patients receiving triplet and quadruplet regimens were younger compared with those receiving monotherapy and doublet regimens (<70 years old: 58% for triplet, 65% for quadruplet vs. 39% for monotherapy, 36% for doublet). Moreover, a higher proportion of patients with stage III disease received triplet (22%) and quadruplet (34%) regimens compared with monotherapy (16%) and doublet regimens (18%).

Exploratory prognostic models were developed and showed that overall, age <70 years, female gender, and treatment at an academic practice were significantly prognostic for longer OS, whereas ECOG >1, ISS disease stages II and III, and high‐risk status were significantly associated with shorter OS (Table 4). Among early transplant recipients, prognostic trends were consistent with the broader cohort, with the exception of age, female gender, and ECOG status of 1, which showed no association. While significant prognostic factors were consistent with the broader cohort in transplant‐ineligible patients (age not applicable), high‐risk status showed no association with OS.

**TABLE 4:** OVerall prognostic model for OS.

Characteristic	HR	95% CI	*p* value
Age at diagnosis			
Age <70 years	—	—	
Age ≥70 years	0.73	0.66, 0.80	<0.001
Received SCT	0.32	0.28, 0.37	<0.001
Practice type			
Community	—	—	
Academic	0.91	0.78, 1.06	0.2
ECOG			
0	—	—	
1	1.43	1.22, 1.68	<0.001
2+	2.17	1.82, 2.57	<0.001
Unknown/not documented	1.54	1.33, 1.77	<0.001
ISS stage			
Stage I	—	—	
Stage II	1.64	1.38, 1.94	<0.001
Stage III	2.28	1.94, 2.68	<0.001
Unknown/not documented	2.28	1.97, 2.65	<0.001
High‐risk status			
No	—	—	
Yes	1.31	1.20, 1.44	<0.001
Year of diagnosis			
2011–2015	—	—	
2016–2019	0.95	0.86, 1.04	0.3

Abbreviations: CI, confidence interval; ECOG, Eastern Cooperative Oncology Group; HR, hazard ratio; ISS, International Staging System; OS, overall survival; SCT, stem cell transplant.

## 4 DISCUSSION

Novel agents for the treatment of MM have recently become available. This retrospective analysis of real‐world prescribing patterns from 6271 patients, diagnosed and treated over the past 9 years, showed that the predominant front‐line regimen for MM was a triplet regimen over the past several years (36% in 2011, 72% in 2019). This trend was evident regardless of disease stage at diagnosis, SCT eligibility according to age or early SCT. Triplet therapy was also more prominently prescribed for patients who received early SCT compared with those who did not. These were younger patients (<70 years) and therefore were likely to have been better candidates for more aggressive therapy. Among the specific classes of agents prescribed as triplet regimens (Table S4), the IMiD + PI + steroid combination was most prevalent in 2019, having increased steadily in use from 2011. This likely reflects the results of the phase 3 trials demonstrating the utility of bortezomib, lenalidomide, and dexamethasone (VRd) in the newly diagnosed setting, both in transplant eligible and non‐eligible patients, with the SWOG S0777 trial results first reported in 2017.^17^, ^18^ The PI + chemotherapy + steroid triplet combination and PI + steroid doublet combination had comparable prescribing patterns, reaching ~20% in 2014–2015 and decreasing to ~10% in 2019, likely related to decreasing use of the bortezomib, cyclophosphamide, and dexamethasone combination. The median time to initiation of second‐line therapy in the entire cohort was almost 4 years, with 28% of patients in the cohort having initiated second‐line therapy by the end of 2019. Patients diagnosed with stage I disease remained in front‐line treatment for longer before receiving second‐line treatment compared with patients with stage II and stage III disease. Of note, the results of our study do not fully reflect the introduction of novel therapies approved for front‐line MM in the 2018 to 2019 time period, specifically the anti‐CD38 monoclonal antibody daratumumab, with the ALCYONE and MAIA trial results first reported in 2018 and 2019, respectively.^19^, ^20^ In the current report, only 32 patients received an anti‐CD38 regimen, with the majority receiving it in 2019. Shorter TTNT in patients treated with the quadruplet regimen likely reflects the use of multidrug combinations in patients presenting with clinically aggressive disease, a feature that cannot be captured accurately in this dataset. Further studies should aim to observe the uptake of these novel therapies as front‐line therapies in the next several years and the impact on the disease trajectory and prognostic implications for MM.

A study comparing a number of MM registries in the US (Connect MM Registry, a large US, multicenter prospective observational cohort study consisting of patients diagnosed and treated at community clinics and academic centers^22^; Surveillance, Epidemiology, and End Results [SEER] database, a population‐based registry^23^; and the National Cancer Database [NCDB], a clinical cancer hospital‐based registry^24^) showed similar baseline distributions in age and male gender with Flatiron Health; however, a more diverse racial distribution in Flatiron Health consistent with SEER and the NCDB was shown compared with the MM Connect Registry. Increasing OS by year of diagnosis in Flatiron Health was also consistent with the Connect MM Registry, SEER, and the NCDB, suggesting that Flatiron Health is representative of baseline and survival trends in the U.S. MM population. Notably, there is a clear stage migration with more patients being initiated on treatment with early‐stage disease, but it is unclear whether this represents the impact of the revised diagnostic criteria and more widespread use of sensitive imaging techniques or an increasing comfort in starting treatment early given the improved tolerability and efficacy of the modern regimens. Additionally, we observed an increased proportion of patients with high‐risk cytogenetics likely reflecting the increasing use of FISH testing and, in particular, the inclusion of the 1q probe in the FISH sets.

The 4‐year survival rate in this study was 65% in 2016 and has not yet been reached in subsequent years up to 2019; however, the time period by transplant eligibility in this study showed similar trends compared to the Paquin et al.^25^ evaluation of consecutive newly diagnosed MM patients studied at the Mayo Clinic. This study evaluated MM patients diagnosed at 65 years of age and younger and had a stem cell harvest within 12 months of diagnosis. The 5‐year OS rate was 76%, which was higher than the 5‐year OS rate for the transplant‐eligible subgroup in the current study (~60%), reflecting the younger age and lower proportion of high‐risk patients included in the transplant‐eligible cohort analyzed by Paquin et al.^25^

Recent analyses of physician preferences and practice patterns for front‐line MM treatment have revealed findings that are generally consistent with this analysis. Schwartz et al.^10^ collected self‐reported physician prescribing preferences for front‐line MM treatment of SCT‐ineligible patients between 2011 and 2014. Doublet regimens were reported to account for approximately half of front‐line preferences (50% in 2011, 52% in 2014), comparable with our findings of 51% in 2011 and 50% in 2014.^10^ Schwartz et al.^10^ also reported increasing use of triplet combination regimens from 22% in 2011 to 41% in 2014, slightly less than our observed prescriptions of 36% in 2011 and 47% in 2014. Song et al.^11^ reported a dramatic increase in prescribing of PI‐containing regimens (bortezomib) from 17% in 2006 to 2007 to 49% in 2013 to 2014, based on their retrospective analysis of U.S. prescription claims. Our analysis also showed notable increases in PI‐containing regimens during this time period. Similarly, Fonseca et al.^12^ reported a shift in front‐line MM treatment patterns to novel agents and associated gains in survival outcomes. Consistent with a study conducted in a similar patient population treated in the community and academic centers in the United States, a similar treatment regimen distribution was shown with higher use of IMiD + PI + steroid in patients receiving early transplant compared to transplant‐ineligible patients and greater use of PI‐containing regimens in the latter compared to the former.^26^

Finally, Chari et al.'s^13^ report of triplet regimen use in patients with newly diagnosed MM also showed decreased use of chemotherapy‐based regimens compared with IMiD + PI + steroid combinations (15% vs. 5% of specific agent–containing regimens). Patients who received the IMiD + PI + steroid regimen were younger, with lower ECOG performance status and earlier disease stage at diagnosis, had longer median TTNT (39 months), and were more likely to have been treated in a community practice setting compared with those receiving a chemotherapy‐based regimen.^13^ In this study, overall median TTNT for triplets, driven by IMiD + PI + steroid, was slightly shorter compared to doublets, driven by IMiD + steroid. This may be due to adverse events or lower tolerability of triplets compared with doublets in front‐line MM.

This analysis should be interpreted with the consideration of certain strengths and limitations. This was a retrospective observational analysis of real‐world prescribing trends from a large sample of the U.S. community‐based care setting, with a small representation (10%) of academic centers. Management of this population consisted of standard‐of‐care treatments and did not include experimental treatment such as those provided in clinical trials. As may be expected with studies based on data extracted from real‐world practice records, certain variables were incomplete, particularly for information related to disease stage and ECOG performance status at diagnosis. This is particularly important in the context of determining transplant eligibility which is often based on a combination of characteristics including age, performance status, comorbidities and patient preference. We used age as a surrogate for transplant eligibility, which though imperfect allows interpretation of the data in the context of transplant trials which have typically used 65 years of age as cutoff for transplant eligibility. Records missing certain information were not included in assessments that required that information, for example, patients without stage information were not included in the analysis of treatment trends by stage at diagnosis. Dimopoulos et al.^27^ found a similar inconsistency of available disease stage and ECOG performance status information in their review of observational studies in patients with relapsed MM. In the definition of high‐risk based on cytogenetic risk markers, gain(1q) in Flatiron Health is assessed by chromosome 1 abnormalities which encompasses gain(1q) and del(1p). However, it has been reported that gains of chromosome 1 (1q) are one of the most common genetic abnormalities in MM.^28^ Gene expression profile (GEP) data are not available in Flatiron Health and del(13) and non‐hyperdiploid karyotype were not assessed in metaphase cytogenetic studies, so these markers were not included in the high‐risk definition for MM.

In conclusion, triplet combination therapy has become the predominantly prescribed treatment regimen for newly diagnosed MM in the U.S., according to the observed prescribing trends. This study provides the most recent assessment of such trends, highlighting a decreasing use of initial doublet regimens since 2011. Patients deemed eligible for SCT, defined in this analysis as patients younger than 70 years, or who received SCT within 12 months of diagnosis were particularly more likely to receive initial triplet therapy compared with those deemed ineligible and/or who did not receive early SCT. The MM treatment landscape has evolved rapidly and continues to do so, offering new options for patients and treating physicians as reflected in the paradigm shift of prescribed treatments observed in this analysis.

## CONFLICT OF INTEREST

S.K. has participated in advisory boards and Independent review committees for Takeda, Celgene, Janssen, Roche, AbbVie, Merck, Sanofi, and KITE (no personal compensation) and has received research support from the institution for clinical trials from Takeda, Celgene, Janssen, Roche, AbbVie, Merck, Sanofi, KITE, and Bristol‐Myers Squibb. M.W., U.O., and W.‐J.H. are employees of Genentech, a member of the Roche Group. A.S. and S.A. are employees of Genesis Research.

## ETHICS APPROVAL

Institutional Review Board approval was obtained prior to study conduct.

## PATIENT CONSENT

Informed consent was waived as this was a non‐interventional study using routinely collected data. The data are de‐identified and subject to obligations to prevent re‐identification and protect patient confidentiality.

## Supporting information

Table S1‐S4

## Data Availability

The data that support the findings of this study have been originated by Flatiron Health, Inc. These de‐identified data may be made available upon request, and are subject to a license agreement with Flatiron Health; interested researchers should contact <DataAccess@flatiron.com>to determine licensing terms.
