# Supplementary material for: Front‐line treatment patterns in multiple myeloma: An analysis of U.S.‐based electronic health records from 2011 to 2019
Source: Cancer Med. 2021 Aug 16;10(17):5866–77. doi: 10.1002/cam4.4137 (PMC8419764; doi:10.1002/cam4.4137)
Supplement: Supplementary file 1 — Table S1‐S4 [file CAM4-10-5866-s001.docx]

**TABLE S1.** MM drug treatment classes included in the analysis

| **Drug class** | **Included agents** (NCCN Guidelines 2011–2019 for primary treatment of MM) | **Description** |
| --- | --- | --- |
| Monoclonal antibodies | Daratumumab  (NCCN v2, v3, 2016–2019) | Induces tumor cell death via classical Fc-dependent immune effector mechanisms, immunomodulation via the removal of CD38+ immunosuppressor cells, and triggering cellular apoptosis via secondary cross-linking.^1-4^ |
|  | Elotuzumab  (NCCN v2, v3, 2016–2019) | Exerts a dual effect by directly activating NK cells and mediating antibody-dependent cellular cytotoxicity through the CD16 pathway.^5^ |
| Immunomodulatory drugs | Lenalidomide  (NCCN v1-3, 2011–2019) | Antiproliferative activity and interferes in a range of MM cell interactions.^6^ |
|  | Thalidomide  (NCCN v1-3, 2011–2019) | Produces many of the same anti-MM effects as lenalidomide; however, these effects are often less potent.^7^ |
|  | Pomalidomide  (NCCN v2, v3, 2017–2019) | Exhibits many of the same immunomodulatory, antiproliferative and interaction-disrupting effects as lenalidomide and thalidomide, often at a greater relative potency.^6^ |
| Proteasome inhibitors (PIs) | Bortezomib  (NCCN v1-3, 2011–2019) | Current-generation PI. Prevents normal functioning of the proteasome by disrupting chymotrypsin-like activity. Promotes activation of 3 distinct apoptotic pathways that target myeloma cells.^8-10^ |
|  | Carfilzomib  (NCCN v2, 3, 2016–2019) | Next-generation PI. Highly specific inhibitor of proteasome chymotrypsin-like activity. Disrupts ubiquitin-proteasome pathway and leads to induction of apoptosis in myeloma cells.^11^ |
|  | Ixazomib  (NCCN v2, v3, 2016–2019) | Next-generation PI. Induces a variety of apoptotic pathways, the endoplasmic reticulum stress response pathway and upregulation of the *miR33b* tumor suppressor gene.^12^ |
| Chemotherapy | Cyclophosphamide  (NCCN v1-3, 2011–2019) | An immunosuppressant and alkylating agent which damages cellular DNA and has significant immunomodulatory activity, affecting several classes of immune cells (including B and T lymphocytes).^13,14^ |
|  | Doxorubicin  (NCCN v1-3, 2011–2019) | A natural anthracycline antibiotic, presumed to interact with DNA through intercalation.^14-16^ |
|  | Melphalan  (NCCN v1-3, 2011–2019) | An alkylating agent which causes linkages between strands of DNA and inhibits DNA and RNA synthesis.^14,17,18^ |
|  | Vincristine  (NCCN v1-3, 2011–2019) | Binds to tubulin causing microtubule depolymerization, metaphase arrest and apoptotic death of cells undergoing mitosis.^19,20^ |
| Histone deacetylase inhibitor | Panobinostat  (NCCN v2, v3, 2016–2019) | Inhibits a broad range of deacetylases. Thought to elicit antitumor activity primarily through epigenetic modulation of gene expression and inhibition of protein mechanism.^21,22^ |
| Corticosteroids | Dexamethasone  (NCCN v1, 2, 3, 2011–2019) | Suspected to induce apoptosis via transactivation through the glucocorticoid response element, transrepression of NF-kappaB, phosphorylation of RAFTK (Pyk2), or induction of Bim.^23^ |
|  | Methylprednisolone  (not explicitly included) |  |
|  | Prednisone  (not explicitly included) |  |
|  | Prednisolone  (not explicitly included) |  |

Abbreviations: DNA, deoxyribonucleic acid; MM, multiple myeloma; NCCN, National Comprehensive Cancer Network; NK, natural killer; PI, proteasome inhibitors; RNA, ribonucleic acid.

**TABLE S2.** Derived staging criteria based on the International Staging System.^24^

| **Stage** | **Criteria** |
| --- | --- |
| I | Serum β_2_-microglobulin <3.5 mg/L and serum albumin ≥3.5 g/dL |
| II | Serum β_2_-microglobulin 3.5 to <5.5 mg/L with any serum albumin level  ***or*** serum β_2_-microglobulin <3.5 mg/L and serum albumin <3.5 g/dL |
| III | Serum β_2_-microglobulin ≥5.5 mg/L |

**TABLE S3.** Median TTNT in transplant-ineligible patients

|  | **Transplant-ineligible** | | | **Received 1L transplant** | | |
| --- | --- | --- | --- | --- | --- | --- |
|  | **N** | **Events** | **Median TTNT (95% CI), months** | **N** | **Events** | **Median TTNT (95% CI), months** |
| **Overall** | 3168 | 752 | 51.2  (47.11‒55.06) | 1162 | 382 | 60.06  (53.65‒67.91) |
| **Number of agents** |  |  |  |  |  |  |
| Monotherapy | 277 | 59 | 44.25  (36.17‒NA) | 19 | 8 | 67.25  (43.53‒NA) |
| Doublet | 1306 | 343 | 49.45  (44.22‒56.15) | 193 | 69 | 59.93  (47.44‒NA) |
| Triplet | 1517 | 329 | 53.36  (49.45‒60.06) | 894 | 284 | 60.98  (53.95‒77.57) |
| Quadruplet | 62 | 18 | 35.32  (30.32‒NA) | 54 | 21 | 38.67  (26.97‒NA) |
| Other | 6 | 3 | 19.35  (12.45‒NA) | 2 | 0 | NA  (NA‒NA) |
| **Treatment regimen** |  |  |  |  |  |  |
| IMiD+PI+ Steroid | 1103 | 217 | 59.63  (51.19‒64.07) | 731 | 210 | 66.27  (57.17‒80.99) |
| Chemo+PI+ Steroid | 354 | 90 | 50.17  (42.51‒67.88) | 158 | 70 | 42.51  (32.43‒NA) |
| IMiD+Steroid | 545 | 121 | 60.19  (54.9‒NA) | 121 | 35 | 60.68  (52.57‒NA) |
| PI+Steroid | 667 | 190 | 42.09  (37.09‒51.32) | 57 | 29 | 38.18  (30.62‒NA) |
| aCD38+PI+ Steroid | 6 | 1 | 13.4  (13.4‒NA) | 1 | 1 | 19.45  (NA‒NA) |
| aCD38+IMiD+Steroid | 8 | 0 | NA | 0 | 0 | NA |
| aCD38+PI+ IMiD+Steroid | 5 | 0 | NA | 0 | 0 | NA |

Abbreviations: CI, confidence interval; Chemo, chemotherapy; IMiD, immunomodulatory drug; NA, not available; PI, proteasome inhibitor; TTNT, time to next treatment.

**TABLE S4.** Specific classes of agents prescribed as triplet regimens.

| **Treatment agent name** | **N = 2387** |
| --- | --- |
| Dexamethasone | 827 (35%) |
| Bortezomib | 712 (30%) |
| Lenalidomide | 640 (27%) |
| Cycophosphamide | 102 (4.3%) |
| Carfilzomib | 45 (1.9%) |
| Daratumumab | 28 (1.2%) |
| Ixazomib | 7 (0.3%) |
| Anastrozole | 6 (0.3%) |
| Clinical Study Drug | 4 (0.2%) |
| Thalidomide | 3 (0.1%) |
| Prednisone | 2 (<0.1%) |
| Abiraterone | 1 (<0.1%) |
| Azacitidine | 1 (<0.1%) |
| Carboplatin | 1 (<0.1%) |
| Exemestane | 1 (<0.1%) |
| Ibrutinib | 1 (<0.1%) |
| Letrozole | 1 (<0.1%) |
| Leuprolide | 1 (<0.1%) |
| Melphalan | 1 (<0.1%) |
| Panobinostat | 1 (<0.1%) |
| Pomalidomide | 1 (<0.1%) |
| Rituximab/Hyaluronidase | 1 (<0.1%) |

**REFERENCES**

1. de Weers M, Tai YT, van der Veer MS, et al. Daratumumab, a novel therapeutic human CD38 monoclonal antibody, induces killing of multiple myeloma and other hematological tumors. *Journal of immunology (Baltimore, Md : 1950).* 2011;186(3):1840-1848.

2. Lammerts van Bueren J, Jakobs D, Kaldenhoven N, et al. Direct in Vitro Comparison of Daratumumab with Surrogate Analogs of CD38 Antibodies MOR03087, SAR650984 and Ab79. *Blood.* 2014;124(21):3474-3474.

3. Overdijk MB, Verploegen S, Bögels M, et al. Antibody-mediated phagocytosis contributes to the anti-tumor activity of the therapeutic antibody daratumumab in lymphoma and multiple myeloma. *mAbs.* 2015;7(2):311-321.

4. Krejcik J, Casneuf T, Nijhof IS, et al. Daratumumab depletes CD38+ immune regulatory cells, promotes T-cell expansion, and skews T-cell repertoire in multiple myeloma. *Blood.* 2016;128(3):384-394.

5. Lonial S, Dimopoulos M, Palumbo A, et al. Elotuzumab Therapy for Relapsed or Refractory Multiple Myeloma. *The New England journal of medicine.* 2015;373(7):621-631.

6. Quach H, Ritchie D, Stewart AK, et al. Mechanism of action of immunomodulatory drugs (IMiDS) in multiple myeloma. *Leukemia.* 2010;24(1):22-32.

7. Stewart AK. Medicine. How thalidomide works against cancer. *Science (New York, NY).* 2014;343(6168):256-257.

8. Lauricella M, Emanuele S, D’Anneo A, et al. JNK and AP-1 mediate apoptosis induced by bortezomib in HepG2 cells via FasL/caspase-8 and mitochondria-dependent pathways. *Apoptosis.* 2006;11(4):607-625.

9. Gu H, Chen X, Gao G, Dong H. Caspase-2 functions upstream of mitochondria in endoplasmic reticulum stress-induced apoptosis by bortezomib in human myeloma cells. *Molecular cancer therapeutics.* 2008;7(8):2298-2307.

10. Voortman J, Resende TP, Abou El Hassan MA, Giaccone G, Kruyt FA. TRAIL therapy in non-small cell lung cancer cells: sensitization to death receptor-mediated apoptosis by proteasome inhibitor bortezomib. *Molecular cancer therapeutics.* 2007;6(7):2103-2112.

11. Demo SD, Kirk CJ, Aujay MA, et al. Antitumor activity of PR-171, a novel irreversible inhibitor of the proteasome. *Cancer research.* 2007;67(13):6383-6391.

12. Tian Z, Zhao J-j, Tai Y-T, et al. Investigational agent MLN9708/2238 targets tumor-suppressor miR33b in MM cells. *Blood.* 2012;120(19):3958-3967.

13. Swan D, Gurney M, Krawczyk J, Ryan AE, O'Dwyer M. Beyond DNA Damage: Exploring the Immunomodulatory Effects of Cyclophosphamide in Multiple Myeloma. *HemaSphere.* 2020;4(2):e350.

14. Gourzones C, Bret C, Moreaux J. Treatment May Be Harmful: Mechanisms/Prediction/Prevention of Drug-Induced DNA Damage and Repair in Multiple Myeloma. 2019;10(861).

15. Taymaz-Nikerel H, Karabekmez ME, Eraslan S, Kırdar B. Doxorubicin induces an extensive transcriptional and metabolic rewiring in yeast cells. *Scientific Reports.* 2018;8(1):13672.

16. Anders CK, Adamo B, Karginova O, et al. Pharmacokinetics and efficacy of PEGylated liposomal doxorubicin in an intracranial model of breast cancer. *PloS one.* 2013;8(5):e61359.

17. Spanswick VJ, Lowe HL, Newton C, et al. Evidence for different mechanisms of 'unhooking' for melphalan and cisplatin-induced DNA interstrand cross-links in vitro and in clinical acquired resistant tumour samples. *BMC cancer.* 2012;12:436.

18. Spanswick VJ, Craddock C, Sekhar M, et al. Repair of DNA interstrand crosslinks as a mechanism of clinical resistance to melphalan in multiple myeloma. *Blood.* 2002;100(1):224-229.

19. Groth-Pedersen L, Ostenfeld MS, Høyer-Hansen M, Nylandsted J, Jäättelä M. Vincristine induces dramatic lysosomal changes and sensitizes cancer cells to lysosome-destabilizing siramesine. *Cancer research.* 2007;67(5):2217-2225.

20. Blajeski AL, Phan VA, Kottke TJ, Kaufmann SH. G(1) and G(2) cell-cycle arrest following microtubule depolymerization in human breast cancer cells. *The Journal of clinical investigation.* 2002;110(1):91-99.

21. Richardson PG, Laubach JP, Lonial S, et al. Panobinostat: a novel pan-deacetylase inhibitor for the treatment of relapsed or relapsed and refractory multiple myeloma. *Expert Review of Anticancer Therapy.* 2015;15(7):737-748.

22. Laubach JP, Moreau P, San-Miguel JF, Richardson PG. Panobinostat for the Treatment of Multiple Myeloma. *Clinical cancer research : an official journal of the American Association for Cancer Research.* 2015;21(21):4767-4773.

23. Sharma S, Lichtenstein A. Dexamethasone-induced apoptotic mechanisms in myeloma cells investigated by analysis of mutant glucocorticoid receptors. *Blood.* 2008;112(4):1338-1345.

24. Greipp PR, San Miguel J, Durie BGM, et al. International staging system for multiple myeloma. *J Clin Oncol*. 2005;23(15):3412-3420.
